# Simultaneous Recognition of Species, Quality Grades, and Multivariate Calibration of Antioxidant Activities for 12 Famous Green Teas Using Mid- and Near-Infrared Spectroscopy Coupled with Chemometrics

**DOI:** 10.1155/2019/4372395

**Published:** 2019-01-02

**Authors:** Haiyan Fu, Ou Hu, Lu Xu, Yao Fan, Qiong Shi, Xiaoming Guo, Wei Lan, Tianming Yang, Shunping Xie, Yuanbin She

**Affiliations:** ^1^The Modernization Engineering Technology Research Center of Ethnic Minority Medicine of Hubei Province, School of Pharmaceutical Sciences, South-Central University for Nationalities, Wuhan 430074, China; ^2^College of Material and Chemical Engineering, Tongren University, Tongren 554300, Guizhou, China; ^3^State Key Laboratory of Green Chemistry-Synthesis Technology, College of Chemical Engineering, Zhejiang University of Technology, Hangzhou 310014, China; ^4^Technology Center, China Tobacco Guizhou Industrial Co., Ltd., Guiyang 550009, Guizhou, China

## Abstract

In this paper, mid- and near-infrared spectroscopy fingerprints were combined to simultaneously discriminate 12 famous green teas and quantitatively characterize their antioxidant activities using chemometrics. A supervised pattern recognition method based on partial least square discriminant analysis (PLSDA) was adopted to classify the 12 famous green teas with different species and quality grades, and then optimized sample-weighted least-squares support vector machine (OSWLS-SVM) based on particle swarm optimization was employed to investigate the quantitative relationship between their antioxidant activities and the spectral fingerprints. As a result, 12 famous green teas can be discriminated with a recognition rate of 100% by MIR or NIR data. However, compared with individual instrumental data, data fusion was more adequate for modeling the antioxidant activities of samples with RMSEP of 0.0065. Finally, the performance of the proposed method was evaluated and validated by some statistical parameters and the elliptical joint confidence region (EJCR) test. The results indicate that fusion of mid- and near-infrared spectroscopy suggests a new avenue to discriminate the species and grades of green teas. Moreover, the proposed method also implies other promising applications with more accurate multivariate calibration of antioxidant activities.

## 1. Introduction

Free radicals (FRs) are defined as atomic or molecular species with unpaired electrons on an otherwise open shell configuration [1]. In recent years, it has been confirmed that FR action is implicated in several chronic and ageing diseases, such as cancer, stroke, heart disease, cataracts, rheumatoid arthritis, and Alzheimer's disease, because FR could damage or cause complete degradation of essential molecules in cells [2, 3]. There is growing evidence that antioxidants could protect the body against the destructive effects of FR [4, 5]. Therefore, antioxidant therapy has emerged as an important approach for maintaining human health and curing diseases.

Consumers are more inclined to take natural antioxidants rather than synthetic antioxidants due to their minimal toxicity. Green tea is considered to be a good source of natural antioxidants [6]. Increasing evidences demonstrate that polyphenolic compounds mainly including (-)-epicatechin (EC), (-)-epigallocatechin (EGC), (-)-epicatechin gallate (ECG), (-)-epigallocatechin gallate (EGCG), and (-)-gallocatechin gallate (GCG) in green teas exhibit antioxidant activity (AA) by adsorbing and neutralizing free radicals, quenching singlet and triplet oxygen, or decomposing peroxides [6–8]. The AA of green tea would differ depending on tea's species and quality grades, etc. AA is not only a criterion for evaluation of medical functions of green tea but also an essential standard for quality monitoring and control in the tea production line. Therefore, it is significant to simultaneously recognize green teas with different species and quality grades and perform qualitative and quantitative analysis of AA for green teas.

Nowadays, wet-chemical analysis such as 2,2-diphenyl-1-picrylhydrazyl (DPPH), 2,2′-azino-bis (3-ethylbenz-thiazoline-6-sulfonic acid) (ABTS), ferric reducing/antioxidant power (FRAP), trolox equivalent antioxidant capacity (TEAC), and oxygen radical absorbance capacity (ORAC) are widely used to measure the antioxidant activity of various food samples [9–11]. However, these methods are time consuming and inconvenient for the applications of online and rapid analysis. With the advantages of being accurate, cost-efficient, rapid, nondestructive, and reagent-free, spectroscopic techniques including mid- and near-infrared (MIR/NIR) spectroscopy have been utilized as alternatives to wet-chemical analysis [12, 13]. In recent years, individual MIR or NIR spectroscopy coupled with multivariate calibration has been successfully used to develop spectra-antioxidant activity relationship models [14–22]. For instance, Hu et al. reports the use of ATR-FT-IR spectroscopy for rapid prediction of antioxidant capacity in chocolate [15], Lucas et al. have been engaged to develop visible-near-infrared reflectance spectroscopy combined with chemometric data analysis methods for predicting total antioxidant capacity in both fresh and freeze-dried cheeses [17]. Li et al. have reported the use of single NIR and MIR spectroscopy for rapid determination of antioxidant activity of *Radix Scutellariae* from different geographical regions [16].

Green tea is a natural healthy product rich in polyphenolic compounds composed of a large number of hydrogenous bonds (i.e. C-H, O-H, and N-H) [23]. Based on molecular vibrations, MIR could represent the absorbance spectra of all chemical bonds in the range from 4000 to 400 cm^−1^ according to the unique correlation of spectral band positions with certain substructures, whereas NIR spectroscopy is within the wavelength range of 800–2500 nm that contains overtones and combinations of fundamental vibrations due to the stretching and bending of hydrogenous bonds [24]. Therefore, the constituents that contribute to antioxidant activity could be characterized by MIR and NIR spectroscopy. For example, Luypaert et al. [25] utilized near-infrared spectroscopy and partial least squares (PLS) to estimate the total antioxidant capacity of green tea. Zhang et al. [26] achieved prediction of the total antioxidant capacity of green tea by a combination of NIR spectroscopy and chemometrics. However, most studies have focused on using NIR spectroscopy and chemometrics for the antioxidant activity analysis of green tea. It is still necessary to evaluate the antioxidant activity of green tea more accurately including also MIR spectroscopy. Moreover, available information about the antioxidant activity of green tea using data fusion of MIR and NIR is scant. Based on the different characteristics of MIR and NIR, the information fusion of MIR and NIR spectra could obtain more comprehensive and reliable description than single spectroscopy to enable more accurate qualitative and quantitative analysis. To the best of our knowledge, combinatory MIR and NIR spectroscopy coupled with multivariate calibration has not been reported for revealing spectra-antioxidant activity relationship in green teas.

In this work, a novel strategy based on the combination of MIR and NIR spectroscopy was developed and validated for the discrimination of 12 famous green teas with different species and quality grades, as well as for quantitative characterization of their antioxidant activity. Because the spectroscopic techniques provide multivariate and nonspecific signals, chemometrics methods for pattern recognition and multivariate calibration were employed to extract most useful and relevant information. This information fusion approach based on MIR and NIR spectroscopy combined with chemometrics was proved to enable reliable classification of green teas and quantitative characterization with high accuracy for spectra-antioxidant activity relationship.

## 2. Materials and Methods

### 2.1. Chemicals and Materials

A total number of twelve famous green tea samples with different species and quality rank were purchased from the representative local tea market, and their detailed information is listed in Table 1. 1,1-diphenyl-2-picrylhydrazyl (DPPH), 2,2'-azinobis (3-ethylbenzothiazoline-6-sulfonic acid) (ABTS), and methanol with analytical grade were obtained from Chinese Medicine Reagent Co., Ltd. Deionized water was collected from a Milli-Q plus purification system (18.25 MΩ·cm).

### 2.2. Sample Preparation and Spectral Acquisition

Each of 12 famous green teas included 10 samples. All green tea samples were crushed into powder and passed through a 200-mesh sieve to ensure homogeneous size of powder particles. The sieved powders were dried under vacuum at 60°C for 24 h and stored in a dryer spare part. For MIR spectroscopy, a Nicolet 6700 FT-IR spectrometer (Thermo Fisher Scientific Inc., USA) was used to collect spectra in the wavelengths ranged from 4000 cm^−1^ to 400 cm^−1^ with a resolution of 4 cm^−1^. A total of 32 scans were accumulated per measurement. Sample scanning was acquired by diffuse reflectance mode. For NIR spectroscopy, 1.0 g powdered sample was weighed accurately and placed into a quartz glass cell for subsequent spectral measurement. NIR spectra were acquired by the diffuse reflectance mode with the use of Antaris II FT-NIR spectrometer (Thermo Electron Co., USA) over the 10000–4000 cm^−1^ range with a resolution of 8 cm^−1^. For each spectrum, the number of scans was set to be 32. The average of three measured spectra of each sample was employed for data processing in this work.

### 2.3. Antioxidant Assay

The sieved green tea powder was subjected to extraction by methyl at a ratio of 1 : 100 (w/v) at 40°C for 20 min and filtered thereafter. The supernatant was prepared for the antioxidant activity assay. The scavenging activities of each green tea were determined with 10 samples by two distinctive antioxidant assays including the DPPH method and the ABTS assay.

In the DPPH method, 90 *μ*L from 1 mM methanol solution of DPPH was separately added into 910 *μ*L methanol diluted green tea extracts (1 : 10). The reaction solutions were blended vigorously and their ultraviolet spectra were subsequently measured at 517 nm after 30-minute standing in the dark at room temperature. Each type of green tea was measured with 10 samples. The scavenging activity (SA) was calculated as below:

(1) $$\begin{array}{c} \text{SA}\left(\%\right)=\left(1-\frac{A_{\text{control}}}{A_{\text{sample}}}\right)\times 100\%. \end{array}$$

In the ABTS assay, 0.15 mM, 0.3 mM, 0.6 mM, and 0.9 mM trolox standards was obtained by appropriate dilution with methanol. The hydrogen peroxide solution was diluted 1000 times by distilled water, and the peroxidase was diluted 10 times with phosphate buffer. The preparation of ABTS solution was performed by adding 152 *μ*L phosphate buffer, 10 *μ*L original ABTS solution, and 8 *μ*L diluted hydrogen peroxide solution. The volume of 170 *μ*L ABTS solution was prepared for the following measurements. The solutions contained 20 *μ*L peroxidase and 10 *μ*L methanol/standard with different concentrations/diluted green tea extract (1 : 50), and 170 *μ*L ABTS solution was measured at 414 nm after incubation for 6 min in the dark at room temperature. The total inhibitory activity of a green tea sample could be calculated according to the equivalent antioxidant capacity (TEAC). In this research, the antioxidant capacity of trolox standard (1 mM) was set at 1 mmol/L.

### 2.4. The Data Fusion of Spectroscopy and Data Processing Method

The raw instrumental data were acquired through above MIR and NIR spectroscopy individually. Data fusion was performed by matrix augmentation. The final MIR-NIR matrix consisted of 3426 variables (1869 MIR variables and 1557 NIR variables) of 120 samples from 12 famous green teas.

In this research, partial least square discriminant analysis (PLSDA) [27], moving window partial least-squares (MWPLS) regression [28, 29], and optimized sample-weighted least-squares support vector machine (OSWLS-SVM) by particle swarm optimization algorithms [30] were used to identify the 12 famous green tea samples with different species and grade levels, select variable regions with higher weights, and construct appropriate spectra-antioxidant activity relationship models, respectively. All data processing programs were written and performed in Matlab software environment.

## 3. Results and Discussion

### 3.1. Analysis of MIR and NIR Spectral Fingerprints

**Figure 1:**
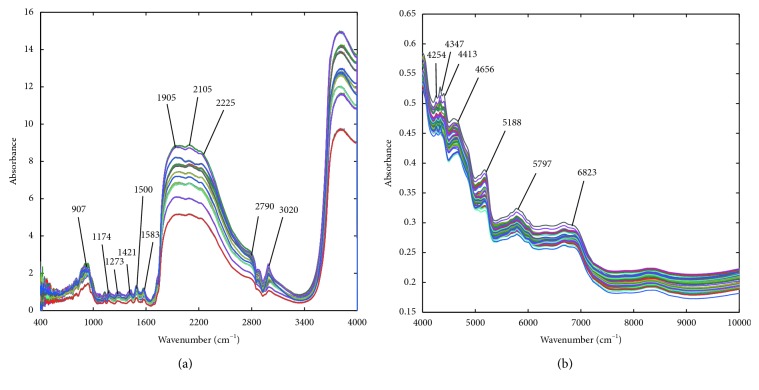
(a) Raw MIR spectra of 12 green tea samples; (b) raw NIR spectra of 12 green tea samples.

The original MIR and NIR spectra of the 12 famous green teas are plotted in Figure 1. The raw MIR or NIR spectra are seriously overlapped and have a poor peak resolution, which makes the accurate assignments of specific peaks very difficult. For ease of peak attributions, chemical bonds are denoted as atom-atom, where an atom can be carbon (C), hydrogen (H), oxygen (O), and nitrogen (N).

The MIR spectra of 12 famous green teas are shown in Figure 1(a), the characteristic absorption peaks can be attributed as follows. In the wavenumber range of 1600 cm^−1^–970 cm^−1^, there are some slight differences in the absorption of MIR spectra, while the absorption spectra of different green teas are quite different in the range of 2790 cm^−1^–1905 cm^−1^. Variations around the peak at 1200 cm^−1^–1900 cm^−1^ can be associated with C-H group and the peak of 1500 cm^−1^–1200 cm^−1^ can be associated with C-O group. The wide scope between 3400 cm^−1^ and 1600 cm^−1^ mainly consists of the overlapping of O-H stretching (3500 cm^−1^–3020 cm^−1^) and various N-H bending and stretching vibrations of amide compounds (3400 cm^−1^–1621 cm^−1^). The asymmetric vibration of CH_2_ at 3020 cm^−1^ can be attributed to tea polyphenols, caffeine, and so on. These components also contribute to O-H stretching vibration.

Figure 1(b) shows NIR spectra of the 12 famous green teas, and the characteristic absorption peaks can be interpreted as follows: the peak at 4254 cm^−1^ is the combination absorbance of C-H symmetric stretching and C-H bending in the phenyl or the second overtone of CH_2_ bending; peaks around 4347 cm^−1^ and 4413 cm^−1^ can be attributed to the combination absorbance of C-H asymmetric stretching and C-H bending, and these peaks may be relevant to tea polyphenols, catechin, and their derivatives; the peak band at 4656 cm^−1^ is due to the combination stretching vibration of C=C, =C-H bands and combination of the base bands of N-H stretching and bending; the peak around 5188 cm^−1^ can be explained as second overtone of C=O stretching bands, first overtone of C-H stretching bands in aromatic rings and combination of the base bands of O-H stretching and bending; and the peak at 5797 cm^−1^ can be responsible for the second overtones of C-H stretching in various groups and peak around 6823 cm^−1^ can be caused by the first overtone of O-H stretch from amino acids and caffeine.

In general, different vibration modes can be attributed to functional groups from chemical components of green teas such as tea polyphenols, amino acids, caffeine, gallic acid, and theobromine. The low spectral resolution was mainly caused by the overlapping of multicomponents, and the shifts and distortions resulted from their interactions. Fortunately, MIR and NIR spectral fingerprints can still reflect characteristics of chemical bonds, and the multivariate variations among samples can provide useful information for classification and calibration. Therefore, chemometric methods are required to develop discriminant model and spectra-antioxidant activity model.

### 3.2. Types and Grades Discrimination of 12 Famous Green Teas by PLSDA

In this work, MIR and NIR spectroscopy coupled with a supervised pattern recognition method based on PLSDA was adopted to identify 12 famous green teas with different species and quality rank. For model coding, a vector *f*_j_ is used to encode category of the j^th^ famous green tea group, in which the j^th^ element is 1 and the other elements are 0. The types of unknown samples can be identified according to the dummy codes of the vectors in the classification matrix. If the maximal element of the i^th^ sample appears at the j^th^ position of the classification vector, the sample will be discriminated as the j^th^ group. Herein, the dummy codes for the twelve groups of green tea samples were defined as G01(1,0,0,0,0,0,0,0,0,0,0,0), G02(0,1,0,0,0,0,0,0,0,0,0,0), G03(0,0,1,0,0,0,0,0,0,0,0,0), G04(0,0,0,1,0,0,0,0,0,0,0,0), G05(0,0,0,0,1,0,0,0,0,0,0,0), G06(0,0,0,0,0,1,0,0,0,0,0,0), G07(0,0,0,0,0,0,1,0,0,0,0,0), G08(0,0,0,0,0,0,0,1,0,0,0,0), G09(0,0,0,0,0,0,0,0,1,0,0,0), G10(0,0,0,0,0,0,0,0,0,1,0,0), G11(0,0,0,0,0,0,0,0,0,0,1,0), G12(0,0,0,0,0,0,0,0,0,0,0,1), which were in line with the orders of famous green tea samples shown in Table 1. Furthermore, the optimal proportion of the training samples was determined according to the best training results. 120 samples of 12 green teas were randomly divided into two sets: a training set that had 88 spectra and a prediction set that contained the remaining 32 spectra. The number of samples used in the training set and prediction set for every green tea are listed in detail in Table 2.

**Figure 2:**
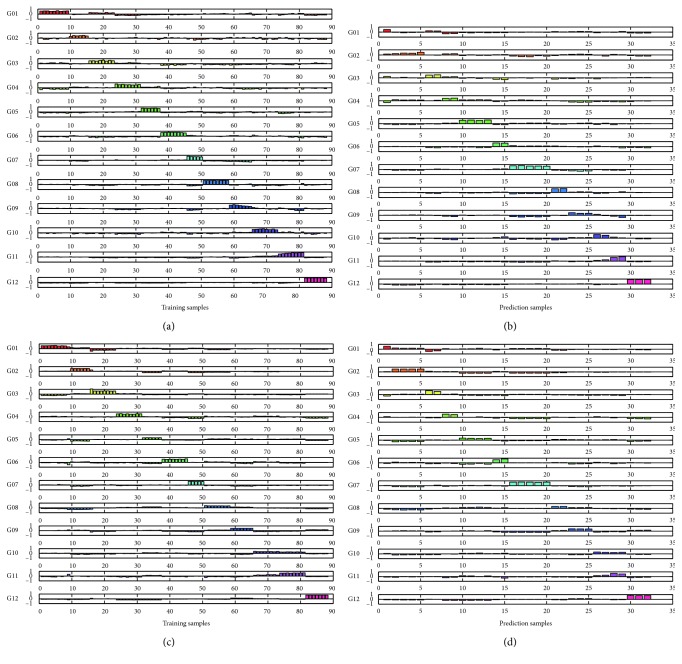
Dummy vectors (G01, G02, G03, G04, G05, G06, G07, G08, G09, G10, G11, G12) of PLSDA for 12 famous green tea samples with different species and grades: (a) 88 training samples from MIR PLSDA model; (b) 32 prediction samples from MIR PLSDA model; (c) 88 training samples from NIR PLSDA model; (d) 32 prediction samples from NIR PLSDA model.

In PLSDA models using the individual MIR and NIR data, the optimal number of PLSDA latent variables was both estimated as 5 by cross validation.  Figure 2 demonstrates the dummy codes of the training sets and prediction sets for the 12 green teas with different species and quality grades. The accuracy rates of training sets and prediction sets for MIR and NIR were all calculated as 100%. To evaluate the accuracy and reliability of discriminant results, analytical figures of merit (FOMs) including sensitivity (SEN) and selectivity (SEL) were calculated. It was found that the PLSDA models constructed by either MIR or NIR data enable classification of the famous green teas with 100% sensitivity and specificity as shown in Table 2.

In this paper, spectral fingerprints of green teas were separately characterized by MIR and NIR spectroscopy in the consideration of antioxidant ingredients chemical information from different characteristic spectroscopic techniques. We also observed 100% discriminant accuracy in training and prediction by single MIR and NIR spectroscopy, indicating individual spectroscopic analytic technology coupled with supervised pattern recognition can obtain satisfactory classification capability to mine tiny chemical differences in famous green teas, and these differences in types and contents will be closely connected with the variations of their antioxidant activities. However, single instrumental analysis is always disadvantaged and restricted by the limited chemical information compared with multidimensional instrumental analysis, so it is imperative to reveal spectra-antioxidant activities by the combinatory MIR-NIR technology, which will be detail discussed in the next section.

### 3.3. Spectra-Antioxidant Activity Relationship Research by OSWLS-SVM

The antioxidant activity values of 12 famous green teas were measured using the reference methods described in the above sections. The values measured using DPPH were in the range from 20.59% to 51.27%. For ABTS, the total antioxidant activity values were characterized by trolox equivalent antioxidant capacity (TEAC) and were in the range of 1.81 to 5.72 mmol/g. The descriptive statistics for these two antioxidant activity indices are presented in Table 3.

In this study, OSWLS-SVM models were developed to separately relate the single MIR, NIR, and combinatory MIR-NIR data to antioxidant activities. The total 120 famous green tea samples were split into a calibration set with 60 samples, a validation set with 30 samples, and a prediction set with 30 samples by DUPLEX method [31]. It is widely recognized that before a multivariate regression model is built, a well-performed variable selection can be helpful to improve the predictive ability of the model. In this paper, moving window partial least-squares (MWPLS) regression as an interval selection method was utilized to extract useful chemical information from MIR, NIR, and fusion of MIR-NIR before the construction of spectra-antioxidant activity relationships by OSWLS-SVM. The individual and combinatory spectroscopic data intervals with lower sums of squared residues (SSR) and less model complexity are selected to reconstruct instrumental response matrices.

**Figure 3:**
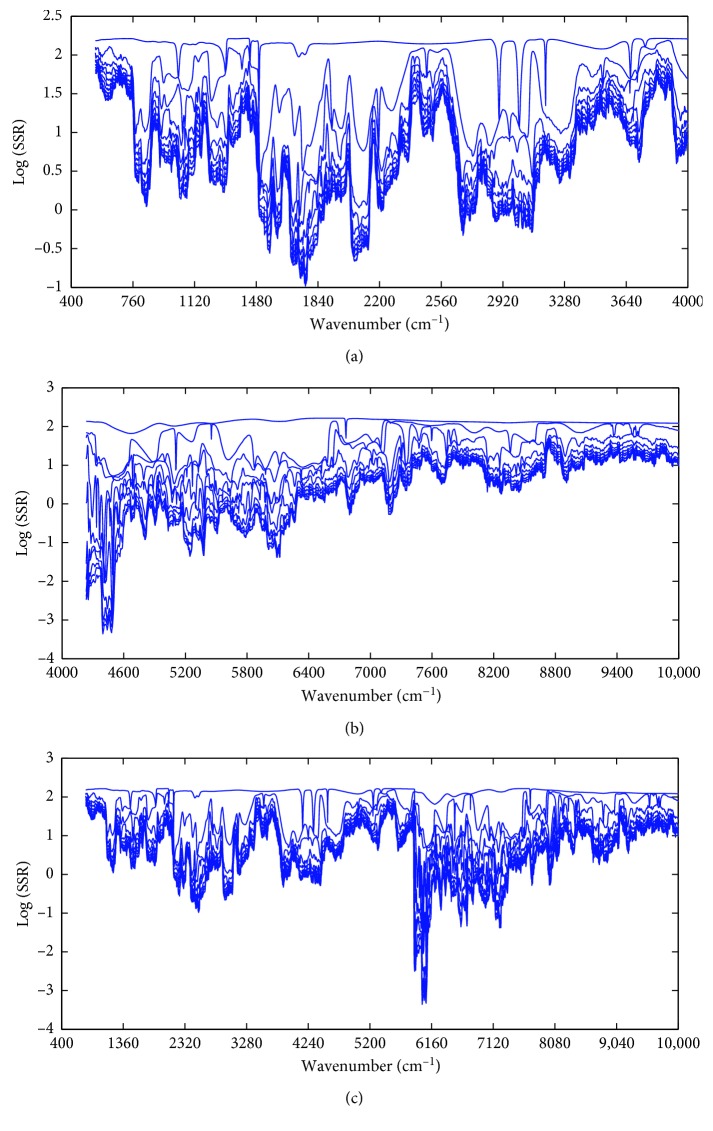
(a) Residue lines obtained by MWPLS for the training sets in MIR spectroscopy; (b) residue lines obtained by MWPLS for the training sets in NIR spectroscopy; (c) residue line obtained by MWPLS for the training sets in the fusion of MIR and NIR spectroscopy.

In the process of variable selection, residue lines obtained by MWPLS for training set with individual and combinatory spectroscopic data are shown in Figure 3. With a window width of 60, the selected regions were located in the data intervals of 1361.50–1496.49 cm^−1^, 1544.70–1718.26 cm^−1^, and 1901.47–2013.32 cm^−1^ for MIR, 4138.49–4281.20 cm^−1^, 4940.73–5187.58 cm^−1^, and 5692.83–6001.39 cm^−1^ for NIR, and 1359.57–1496.49 cm^−1^, 1538.92–1776.12 cm^−1^, 1887.97–2021.03 cm^−1^, 4142.35–4277.34 cm^−1^, 4936.88–5295.57 cm^−1^, and 5681.26–6039.96 cm^−1^ for combinatory data. These intervals could achieve the least complexity and less SSR and were subsequently processed by OSWLS-SVM.

**Figure 4:**
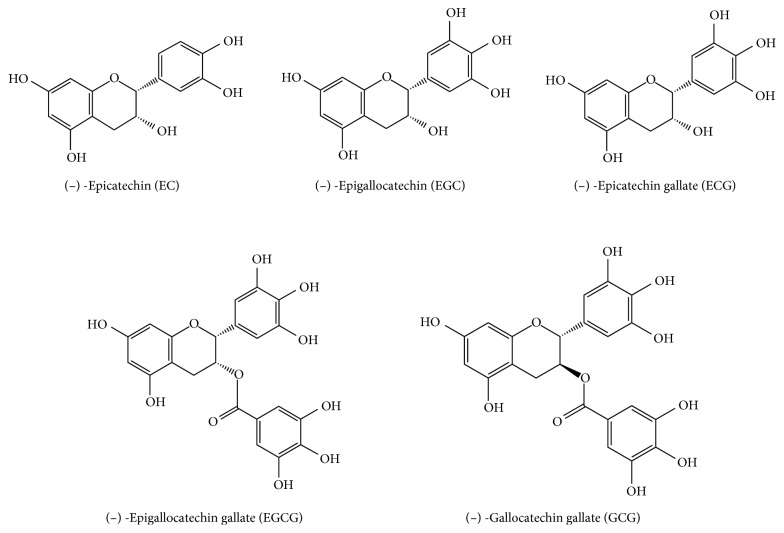
Chemical structures of five representative polyphenols.

Tea is particularly rich in polyphenols including (-)-epicatechin (EC), (-)-epigallocatechin (EGC), (-)-epicatechin gallate (ECG), (-)-epigallocatechin gallate (EGCG), and (-)-gallocatechin gallate (GCG), which act as antioxidants in vitro and in vivo by scavenging reactive oxygen and nitrogen species and chelating redox-active transition metal ions [32]. Chemical structures of some representative polyphenols in green teas are shown in Figure 4. In addition, tea polysaccharide conjugate (TPC) was also proved to have potent antioxidant activity [33]. As seen in Figure 4, polyphenols contained benzene ring, ester group, phenolic hydroxyl group, and the selected wavelength of 1359.57–1496.49 cm^−1^, 1538.92–1776.12 cm^−1^, 1887.97–2021.03 cm^−1^, 4142.35–4277.34 cm^−1^, 4936.88–5295.57 cm^−1^, and 5681.26–6039.96 cm^−1^ by MWPLS can be separately associated with saturated C-H bending vibration; carbon-carbon double bond from aromatic ring and carbanyl group; C-H symmetric stretching and C-H bending in the phenyl or second overtone of CH_2_ bending; second overtone of C=O stretching bands, first overtone of C-H stretching bands in aromatic rings, and combination of the base bands of O-H stretching and bending; and the second overtones of C-H stretching in various groups. Therefore, the selected variable information based on MIR or NIR could adequately reflect the chemical structures from antioxidant ingredients, making the OSWLS-SVM models more interpretable and reasonable.

**Figure 5:**
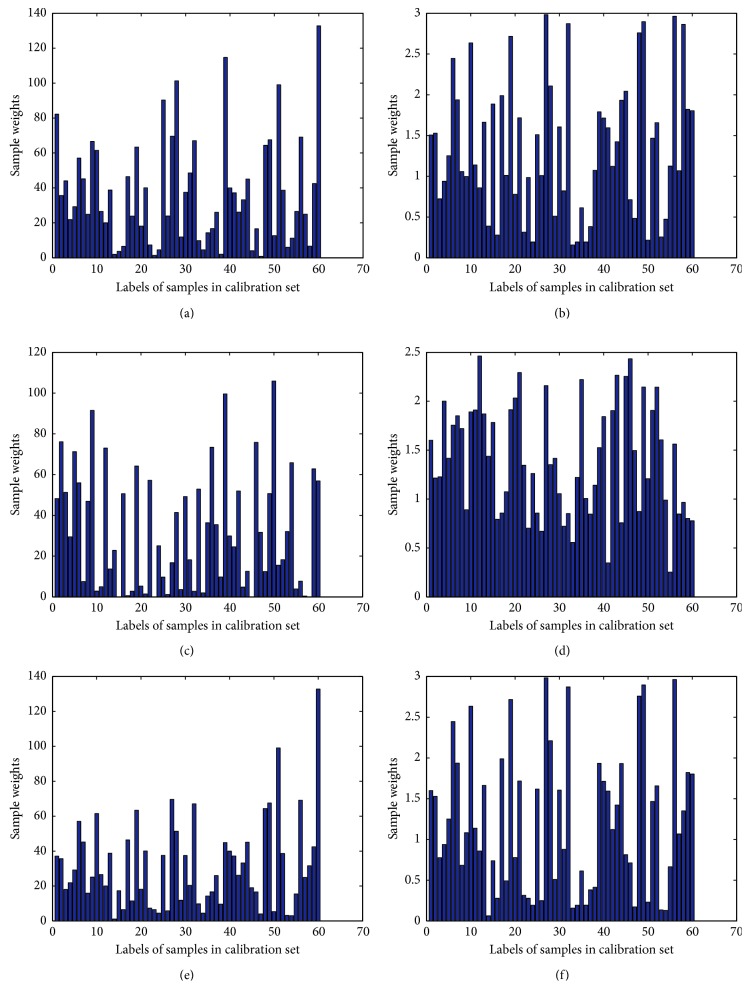
Sample weights after a 70-cycle PSO search for the (a) DPPH scavenging activity characterized by MIR, (b) ABTS scavenging activity characterized by MIR, (c) DPPH scavenging activity characterized by NIR, (d) ABTS scavenging activity characterized by NIR, (e) DPPH scavenging activity characterized by fusion of MIR and NIR, (f) ABTS scavenging activity characterized by fusion of MIR and NIR using OSWLS-SVM.

For OSWLS-SVM, a 70-cycle PSO was carried out to search for the sample weights and hyperparameters minimizing the objective function. The estimated sample weights are shown in Figure 5. It can be seen that most of the samples are high weighted, and useful information carried by these samples would be retained for prediction. The prediction results of antioxidant activities by using the DPPH and ABTS methods based on OSWLS-SVM are summarized in Tables 4 and 5, respectively. These results demonstrated that spectroscopic technology coupled with OSWLS-SVM could reveal potential connection between spectral fingerprints and antioxidant activity and accurately predict the antioxidant activities in green teas.

The recovery was determined by the ratio of predicted radical scavenging activity value to the true radical scavenging activity value. For DPPH scavenging activity, the average predicted recoveries gained from MIR, NIR, and spectroscopic fusion data were 99.8 ± 2.5, 99.7 ± 2.4, and 100.1 ± 1.7%, respectively. For ABTS radical scavenging activity, the average predicted recoveries gained from MIR, NIR, and spectroscopic fusion data were 99.7 ± 1.0, 99.9 ± 0.8, and 100.0 ± 0.4%, respectively. Moreover, the root-mean-squared error of the calibration (RMSEC) in the calibration set and prediction root-mean-squared error (RMSEP) of samples in the prediction set were used to evaluate the accuracy of calibration models. The model with spectroscopic fusion data can obtain the smallest prediction root-mean-squared error, indicating that combinatory spectroscopic model is better than the models using individual data. Meanwhile, it was demonstrated that the OSWLS-SVM model was effective.

**Figure 6:**
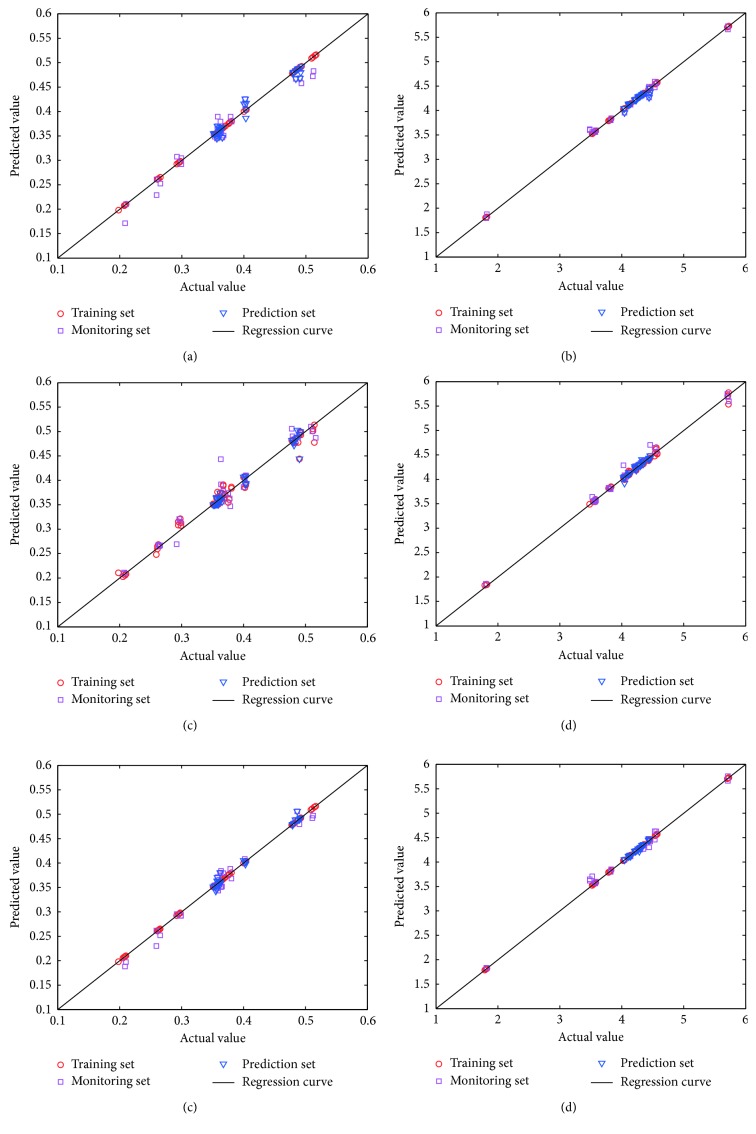
Correlation plots of the predicted antioxidant activities obtained by OSWLS-SVM versus the actual antioxidant activities in 12 famous green teas: (a) DPPH scavenging activity estimated by MIR model; (b) ABTS radical scavenging activity estimated by the NIR model; (c) DPPH scavenging activity estimated by the MIR model; (d) ABTS radical scavenging activity estimated by NIR model; (e) DPPH scavenging activity estimated by the spectroscopic data fusion model; (f) ABTS radical scavenging activity estimated by the spectroscopic data fusion model.

In order to compare prediction performance of MIR, NIR, and MIR-NIR data, the predicted antioxidant activities versus the actual antioxidant activities for the 12 famous green teas are shown in Figure 6. For OSWLS-SVM model for DPPH using MIR, there's slight deviation between experimental values and prediction values in prediction set, but significant deviation exists in monitoring set, indicating that single MIR information could not fully reflect DPPH scavenging activity despite the high correlation. For the model of NIR for DPPH, some prediction values were not very close to actual values in training set; in addition, predictions of several monitoring samples slightly deviate from the actual values for both DPPH and ABTS. NIR also seems to be insufficient in characterizing all the potential antioxidant chemical ingredients. For OSWLS-SVM model using data fusion, the prediction values were very close to actual values. These results confirmed that the combinatory spectroscopic method coupled with OSWLS-SVM was more effective than the single spectroscopic method. The results in Figure 6 also indicate that spectra-antioxidant activity relationship models for ABTS were slightly better than the models for DPPH because DPPH and ABTS radicals have different sensitivity to taste ingredients.

The performance of the proposed method was also evaluated and validated by some statistical parameters. A *t*-test was performed to compare the actual and predicted antioxidant activities estimated by the DPPH and ABTS methods (Tables 4 and 5). Degrees of freedom *n* is 29, and confidence level is 95%, for MIR with DPPH method *t* = 0.0841 < *t*_0.05_^29^_,_ MIR with ABTS method *t* = 0.4615 < *t*_0.05_^29^, NIR with DPPH method *t* = 0.0820 < *t*_0.05_^29^, NIR with ABTS method *t* = 0.0634 < *t*_0.05_^29^, for combinatory spectroscopy with DPPH method *t* = 0.0441 < t_0.05_^29^, combinatory spectroscopy with ABTS method *t* = 0.0054 < *t*_0.05_^29^_._ The results indicate that there's no significant difference between actual and predicted antioxidant activities. Additionally, data fusion can obtain improved capacity to directly predict antioxidant activities of famous green teas.

**Figure 7:**
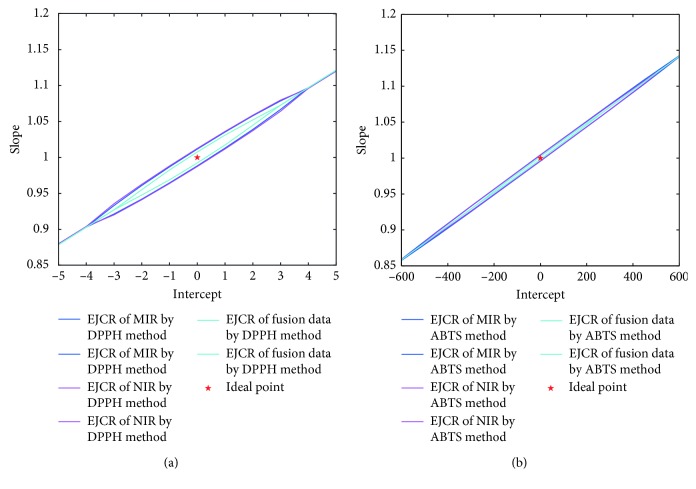
(a) EJCRs for DPPH scavenging activity by applying E-nose, E-tongue, and spectroscopic fusion data; (b) EJCRs for ABTS radical scavenging activity by applying E-nose, E-tongue, and spectroscopic fusion data. The pentacle (★) indicates the ideal points (0, 1).

To further compare the accuracy of individual and combinatory spectroscopy, the experimental antioxidant activities were linearly regressed against the predicted antioxidant activities. The calculated intercept and slope were compared with their ideal values (0 and 1) based on the elliptical joint confidence region (EJCR) test [34]. The EJCR test has been widely used and considered to be an effective and reliable method to evaluate the accuracy of result [35–38].  Figure 7 gives the results of EJCR tests for DPPH and ABTS methods. With the ideal point (0,1) signed with a pentacle (★) lying in the center, the elliptic size of combinatory spectroscopy was found to be smallest and NIR had the biggest elliptic size, indicating that the performance of the OSWLS-SVM model based on combinatory spectroscopic data was better than those of individual spectroscopic data. The results further confirm that spectroscopic fusion strategy coupled with OSWLS-SVM could more sufficiently explain the relationship between overall chemical information of ingredients and antioxidant activity.

## 4. Conclusions

In this study, we developed a novel and effective alternative for the simultaneous recognition of the species and grades of 12 famous green teas and for the multivariate calibration of antioxidant activities of green teas based on MIR and NIR spectroscopy coupled with chemometrics. The 12 famous green teas could be successfully classified with 100% recognition rate using MIR or NIR spectroscopy individually coupled with PLSDA. Furthermore, individual MIR and NIR as well as combinatory spectroscopic data were subsequently related to their antioxidant activities by OSWLS-SVM. The results confirmed the advantages of data fusion of MIR and NIR in comprehensive and reliable characterization of chemical components in green teas. Furthermore, OSWLS-SVM was proved to be an effective approach to characterize the potential relationship between spectroscopic data and antioxidant activities, opening a new avenue for rapidly and more accurately estimating antioxidant activities in other foodstuffs.

## Figures and Tables

**Table 1:** The detailed information of green teas.

Group code	Famous green tea samples	Quality rank
G01	Biluochun	Super second grade
G02	Biluochun	Super grade
G03	Zuyeqing	Lundao grade
G04	Zuyeqing	Qingyuxin grade
G05	Zuyeqing	Pinwei grade
G06	Duyunmaojian	Super grade
G07	Duyunmaojian	First grade
G08	Duyunmaojian	Super first grade
G09	Yunwumaojian	Super grade
G10	Juansumaofeng	First grade
G11	Xinyangmaojian	Super grade
G12	Xinyangmaojian	First grade

**Table 2:** FOMs of MIR and NIR models in 12 famous green teas by the PLSDA method.

Group code	Training set		Prediction set		MIR		NIR	
	Number	Sample	Number	Sample	SEN	SEL	SEN	SEL
G01	9	1^st^–9^th^	1	1^st^	1.00	1.00	1.00	1.00
G02	6	10^th^–15^th^	4	2^nd^–5^th^	1.00	1.00	1.00	1.00
G03	8	16^th^–23^th^	2	6^th^–7^th^	1.00	1.00	1.00	1.00
G04	8	24^th^–31^th^	2	8^th^–9^th^	1.00	1.00	1.00	1.00
G05	6	32^th^–37^th^	4	10^th^–13^th^	1.00	1.00	1.00	1.00
G06	8	38^th^–45^th^	2	14^th^–15^th^	1.00	1.00	1.00	1.00
G07	5	46^th^–50^th^	5	16^th^–20^th^	1.00	1.00	1.00	1.00
G08	8	51^th^–58^th^	2	21^th^–22^th^	1.00	1.00	1.00	1.00
G09	7	59^th^–65^th^	3	23^th^–25^th^	1.00	1.00	1.00	1.00
G10	8	66^th^–73^th^	2	26^th^–27^th^	1.00	1.00	1.00	1.00
G11	8	74^th^–81^th^	2	28^th^–29^th^	1.00	1.00	1.00	1.00
G12	7	82^th^–88^th^	3	30^th^–32^th^	1.00	1.00	1.00	1.00

**Table 3:** The calculation of antioxidant activities from 12 famous green teas by DPPH and ABTS.

Group code	DPPH scavenging activity		ABTS radical scavenging activity	
	Average (%)	RSD	Average (TEAC)	RSD
G01	36.19 ± 0.28	0.77	4.45 ± 0.01	0.25
G02	36.10 ± 0.26	0.73	4.12 ± 0.02	0.36
G03	48.06 ± 0.23	0.47	4.04 ± 0.01	0.18
G04	40.26 ± 0.15	0.37	4.22 ± 0.01	0.22
G05	48.99 ± 0.25	0.51	4.34 ± 0.02	0.35
G06	35.52 ± 0.32	0.90	4.28 ± 0.01	0.35
G07	20.59 ± 0.47	2.30	1.81 ± 0.01	0.76
G08	29.60 ± 0.28	0.93	3.55 ± 0.02	0.56
G09	37.78 ± 0.19	0.51	3.81 ± 0.02	0.42
G10	36.68 ± 0.25	0.68	4.55 ± 0.02	0.35
G11	51.27 ± 0.30	0.58	5.72 ± 0.01	0.16
G12	26.24 ± 0.24	0.93	3.55 ± 0.04	1.18

**Table 4:** The predictions of DPPH scavenging activities by OSWLS-SVM.

Prediction samples	Actual DPPH scavenging activity (%)			Recoveries (%)		
	MIR	NIR	Fusion data	MIR	NIR	Fusion data
1	36.07	36.07	36.07	97.1	100.7	100.0
2	36.53	36.23	36.23	100.0	100.3	100.0
3	36.07	36.53	35.80	101.6	97.5	103.5
4	36.53	36.07	36.23	94.9	101.3	104.9
5	35.65	36.23	35.65	98.2	103.2	97.3
6	36.16	36.53	36.16	100.0	99.8	100.0
7	36.34	35.65	36.16	100.0	101.8	100.0
8	36.16	36.16	36.16	100.0	100.1	98.2
9	35.65	35.65	35.65	96.8	102.4	99.2
10	36.34	36.16	36.34	101.3	99.1	99.2
11	36.16	47.80	36.16	100.0	100.8	100.0
12	47.92	48.06	47.92	100.0	100.2	100.0
13	48.38	48.38	48.38	100.0	100.2	100.9
14	48.06	48.06	47.92	100.0	99.4	99.6
15	48.38	48.17	48.17	96.5	98.0	100.0
16	40.02	40.02	40.02	103.9	101.6	101.2
17	40.37	40.37	40.37	100.0	97.5	100.0
18	40.32	40.32	40.32	95.8	101.4	98.6
19	40.21	40.21	40.21	105.8	100.3	99.6
20	40.37	40.37	40.37	103.3	96.8	100.0
21	48.67	48.67	48.67	100.0	103.3	103.9
22	49.04	49.04	49.04	95.5	100.6	99.7
23	49.16	48.67	49.16	97.6	100.2	100.0
24	48.81	49.04	48.81	100.0	90.5	100.0
25	49.27	49.16	49.27	100.0	101.6	100.0
26	35.11	35.52	35.11	101.0	98.7	100.0
27	35.52	35.73	35.52	100.0	98.3	96.4
28	35.92	35.92	35.92	100.0	97.6	100.0
29	35.32	35.11	35.32	100.0	99.6	100.0
30	35.73	35.32	35.73	103.5	99.1	101.7
Correlation coefficient				1.0000	0.9904	1.0000
Regression equation				*y* = 0.9998*x* + 0.0001	*y* = 0.9538*x* + 0.0173	*y* = 0.9998*x* + 0.0001
Average recoveries (%)				99.8 ± 2.5	99.7 ± 2.4	100.1 ± 1.7
RMSEC^a^				5.7261 × 10^−5^	0.0122	6.4662
RMSEP^b^				0.0100	0.0108	0.0065
T(*t*-test)t_0.05_^29^ = 2.045				0.0841 < t_0.05_^29^	0.0820 < t_0.05_^29^	0.0441 < t_0.05_^29^

^a^RMSEC denotes calibration root-mean-squared error, ^b^RMSEP denotes prediction root-mean-squared error.

**Table 5:** The predictions of ABTS scavenging activities by OSWLS-SVM.

Prediction samples	Actual ABTS radical scavenging activity (TEAC)			Recoveries (%)		
	MIR	NIR	Fusion data	MIR	NIR	Fusion data
1	4.44	4.44	4.44	97.4	99.4	100.0
2	4.46	4.44	4.44	100.0	99.8	100.0
3	4.44	4.46	4.43	96.1	100.5	100.4
4	4.46	4.44	4.44	98.2	99.6	100.7
5	4.11	4.44	4.11	100.5	99.2	100.2
6	4.11	4.46	4.11	100.0	99.5	100.0
7	4.12	4.11	4.14	100.0	100.7	100.0
8	4.14	4.14	4.14	100.0	99.8	99.3
9	4.11	4.11	4.11	100.5	99.8	100.3
10	4.12	4.11	4.12	100.2	99.4	100.0
11	4.14	4.03	4.14	100.0	100.2	100.0
12	4.04	4.04	4.04	100.0	99.7	100.0
13	4.04	4.04	4.04	100.0	100.3	100.1
14	4.04	4.04	4.04	100.0	99.7	100.2
15	4.04	4.04	4.04	97.8	97.0	100.0
16	4.21	4.21	4.21	100.1	101.2	100.3
17	4.22	4.22	4.22	100.0	100.0	100.0
18	4.23	4.23	4.23	99.8	100.4	99.9
19	4.22	4.22	4.22	100.2	100.4	100.1
20	4.23	4.23	4.23	99.5	98.8	100.0
21	4.33	4.33	4.33	100.0	101.8	100.3
22	4.35	4.35	4.35	99.7	100.7	99.9
23	4.36	4.33	4.36	99.5	100.4	100.0
24	4.33	4.35	4.33	100.0	99.2	100.0
25	4.36	4.36	4.36	100.0	100.5	100.0
26	4.27	4.28	4.27	100.4	100.4	100.0
27	4.28	4.29	4.28	100.0	99.8	98.4
28	4.30	4.30	4.30	100.0	99.4	100.0
29	4.27	4.27	4.27	100.0	100.3	100.0
30	4.29	4.27	4.29	99.8	100.4	99.7
Correlation coefficient				1.0000	0.9990	1.0000
Regression equation				*y* = 1.0000*x* + 3.6935 × 10^−6^	*y* = 0.9860*x* + 0.0565	*y* = 1.0000*x* + 2.7420 × 10^−6^
Average recoveries (%)				99.7 ± 1.0	99.9 ± 0.8	100.0 ± 0.4
RMSEC^a^				1.1315 × 10^−6^	0.0394	7.6170 × 10^−7^
RMSEP^b^				0.0447	0.0347	0.0155
T(*t*-test)t_0.05_^29^ = 2.045				0.4615 < t_0.05_^29^	0.0634 < t_0.05_^29^	0.0054 < t_0.05_^29^

^a^RMSEC denotes calibration root-mean-squared error; ^b^RMSEP denotes prediction root-mean-squared error.

## Data Availability

Our data are available on request by interested readers.

## References

[1] Wang C., El-Shetehy M., Shine M. B. (2014). Free radicals mediate systemic acquired resistance. *Cell Reports*.

[2] Sharma N. (2014). Free radicals, antioxidants and disease. *Biology and Medicine*.

[3] Valko M., Jomova K., Rhodes C. J., Kuča K., Musílek K. (2015). Redox- and non-redox-metal-induced formation of free radicals and their role in human disease. *Archives of Toxicology*.

[4] Chen Q., Guo Z., Zhao J., Quyang Q. (2012). Comparisons of different regressions tools in measurement of antioxidant activity in green tea using near infrared spectroscopy. *Journal of Pharmaceutical and Biomedical Analysis*.

[5] Moukette B. M., Pieme C. A., Njimou J. R., Biapa C. P., Marco B., Ngogang J. Y. (2015). In vitro antioxidant properties, free radicals scavenging activities of extracts and polyphenol composition of a non-timber forest product used as spice: monodora myristica. *Biological Research*.

[6] Hae-Suk K., Quon M. J., Jeong -A. K. (2014). New insights into the mechanisms of polyphenols beyond antioxidant properties; lessons from the green tea polyphenol, epigallocatechin 3-gallate. *Redox Biology*.

[7] Afzal M., Safer A. M., Menon M. (2015). Green tea polyphenols and their potential role in health and disease. *Inflammopharmacology*.

[8] Karim A. J., Dalai D. R. (2014). *Green Tea: A Review on its Natural Anti-Oxidant Therapy and Cariostatic Benefits*.

[9] Gülçin İ. (2011). Antioxidant activity of food constituents: an overview. *Archives of Toxicology*.

[10] Kujawska M., Ewertowska M., Ignatowicz E. (2016). Evaluation of safety and antioxidant activity of yellow tea (*Camellia sinensis*) extract for application in food. *Journal of Medicinal Food*.

[11] Samydurai P. (2012). Nutritional assessment, polyphenols evaluation and antioxidant activity of food resource plant Decalepis hamiltonii Wight & Arn. *Journal of Applied Pharmaceutical Science*.

[12] Karoui R., Downey G., Blecker C. (2010). Mid-infrared spectroscopy coupled with chemometrics: a tool for the analysis of intact food systems and the exploration of their molecular Structure−Quality relationships − a review. *Chemical Reviews*.

[13] Luypaert J., Massart D. L., Vander Heyden Y. (2007). Near-infrared spectroscopy applications in pharmaceutical analysis. *Talanta*.

[14] Chen Q., Guo Z., Zhao J., Ouyang Q. (2012). Comparisons of different regressions tools in measurement of antioxidant activity in green tea using near infrared spectroscopy. *Journal of Pharmaceutical and Biomedical Analysis*.

[15] Hu Y., Pan Z. J., Liao W. (2016). Determination of antioxidant capacity and phenolic content of chocolate by attenuated total reflectance-Fourier transformed-infrared spectroscopy. *Food Chemistry*.

[16] Li H., He J., Li F. (2015). Application of NIR and MIR spectroscopy for rapid determination of antioxidant activity of Radix Scutellariae from different geographical regions. *Phytochemical Analysis*.

[17] Lucas A., Andueza D., Rock E., Martin B. (2008). Prediction of dry matter, fat, pH, vitamins, minerals, carotenoids, total antioxidant capacity, and color in fresh and freeze-dried cheeses by visible-near-infrared reflectance spectroscopy. *Journal of Agricultural and Food Chemistry*.

[18] Ma L., Zhang Z., Zhao X., Zhang S., Lu H. (2016). The rapid determination of total polyphenols content and antioxidant activity in Dendrobium officinale using near-infrared spectroscopy. *Analytical Methods*.

[19] Niero G., Penasa M., Currò S. (2016). Development and validation of a near infrared spectrophotometric method to determine total antioxidant activity of milk. *Food Chemistry*.

[20] Rahman M. M. (2014). Orthogonal partial least squares model for rapid prediction of antioxidant activity of by fourier transform infrared spectroscopy. *Analytical Letters*.

[21] Wu D., Chen J., Lu B., Xiong L., He Y., Zhang Y. (2012). Application of near infrared spectroscopy for the rapid determination of antioxidant activity of bamboo leaf extract. *Food Chemistry*.

[22] Wu Z., Xu E., Long J. (2015). Rapid measurement of antioxidant activity and γ-aminobutyric acid content of Chinese rice wine by fourier-transform near infrared spectroscopy. *Food Analytical Methods*.

[23] Ridder L., van der Hooft J. J. J., Verhoeven S., de Vos R. C. H., Bino R. J., Vervoort J. (2013). Automatic chemical structure annotation of an LC-MSn based metabolic profile from green tea. *Analytical Chemistry*.

[24] Bertrand D., Dufour E. (2012). *Book Analysis by NIR and MIR Spectroscopy*.

[25] Luypaert J., Zhang M. H., Massart D. L. (2003). Feasibility study for the use of near infrared spectroscopy in the qualitative and quantitative analysis of green tea, *Camellia sinensis* (L.). *Analytica Chimica Acta*.

[26] Zhang M. H., Luypaert J., Fernández Pierna J. A., Xu Q. S., Massart D. L. (2004). Determination of total antioxidant capacity in green tea by near-infrared spectroscopy and multivariate calibration. *Talanta*.

[27] Fan Y., Liu L., Sun D. (2016). “Turn-off” fluorescent data array sensor based on double quantum dots coupled with chemometrics for highly sensitive and selective detection of multicomponent pesticides. *Analytica Chimica Acta*.

[28] Fu H.-Y., Huan S.-Y., Xu L. (2017). Moving window partial least-squares discriminant analysis for identification of different kinds of bezoar samples by near infrared spectroscopy and comparison of different pattern recognition methods. *Journal of Near Infrared Spectroscopy*.

[29] Jiang J.-H., Berry R. J., Siesler H. W., Ozaki Y. (2002). Wavelength interval selection in multicomponent spectral analysis by moving window partial least-squares regression with applications to mid-infrared and near-infrared spectroscopic data. *Analytical Chemistry*.

[30] Yu H.-Y., Wu H.-L., Zou H.-Y. (2010). Automatic configuration of optimized sample-weighted least-squares support vector machine by particle swarm optimization for multivariate spectral analysis. *Analytical Methods*.

[31] Snee R. D. (1977). Validation of regression models: methods and examples. *Technometrics*.

[32] Frei B., Higdon J. V. (2003). Antioxidant activity of tea polyphenols in vivo: evidence from animal studies. *Journal of Nutrition*.

[33] Chen H., Zhang M., Qu Z., Xie B. (2008). Antioxidant activities of different fractions of polysaccharide conjugates from green tea (*Camellia Sinensis*). *Food Chemistry*.

[34] González A. G., Herrador M. A., Asuero A. G. (1999). Intra-laboratory testing of method accuracy from recovery assays. *Talanta*.

[35] Li Y.-N., Wu H.-L., Qing X.-D. (2010). Quantitative analysis of triazine herbicides in environmental samples by using high performance liquid chromatography and diode array detection combined with second-order calibration based on an alternating penalty trilinear decomposition algorithm. *Analytica Chimica Acta*.

[36] Liu Z., Wu H.-L., Xie L.-X. (2017). Chemometrics-enhanced liquid chromatography-full scan-mass spectrometry for interference-free analysis of multi-class mycotoxins in complex cereal samples. *Chemometrics and Intelligent Laboratory Systems*.

[37] Nie J., Wu H., Wang X., Zhang Y., Zhu S., Yu R. (2008). Determination of testosterone propionate in cosmetics using excitation-emission matrix fluorescence based on oxidation derivatization with the aid of second-order calibration methods. *Analytica Chimica Acta*.

[38] Vosough M., Eshlaghi S. N., Zadmard R. (2015). On the performance of multiway methods for simultaneous quantification of two fluoroquinolones in urine samples by fluorescence spectroscopy and second-order calibration strategies. *Spectrochimica Acta Part A: Molecular and Biomolecular Spectroscopy*.

